# A fatal case of neonatal viral sepsis caused by human parainfluenza virus type 3

**DOI:** 10.1186/s12985-023-02141-9

**Published:** 2023-10-27

**Authors:** Xiangpeng Chen, Hong Wang, Qi Li, Yujie Qi, Fei Li, Wenwen He, Qiushi Wang, Fei Jin, Yanqing Guo, Mingyan Hei, Zhengde Xie

**Affiliations:** 1 Beijing Key Laboratory of Pediatric Respiratory Infection Diseases, Key Laboratory of Major Diseases in Children, Ministry of Education, National Clinical Research Center for Respiratory Diseases, Research Unit of Critical Infection in Children, Laboratory of Infection and Virology, Beijing Children’s Hospital, Chinese Academy of Medical Sciences, Beijing Pediatric Research Institute, Capital Medical University, National Center for Children’s Health, No. 56 Nan-li-shi Road, Xicheng District, Beijing, 2019RU016, 100045 China; 2 Department of Neonatology, Neonatal Center, Beijing Children’s Hospital, Capital Medical University, National Center for Children’s Health, Beijing, 100045 China; 3 Infection Business Unit, Tianjin Novogene Med LAB Co., Ltd, Tianjin, 301700 China

**Keywords:** Sepsis, Septic shock, Human parainfluenza virus

## Abstract

**Background:**

Sepsis is a systemic inflammatory response syndrome caused by severe infection in children, but cases of sepsis associated with human parainfluenza virus (HPIV) have been rarely reported in newborns.

**Case presentation:**

We report a case of HPIV-3 positive full-term newborn admitted to the Neonatal Intensive Care Unit of Beijing Children’s Hospital due to hematuria, gloomy spirit, inactivity and loss of appetite for 6 h. He had septic shock when he arrived the Accident & Emergency Department requiring immediate intubation and mechanical ventilation. Intravenous antibiotics were started. He had completely negative response to all anti-shock treatments including fluid resuscitation and vasopressor supports, and died 14 h later. Viral nucleic acid detection and metagenomic next-generation sequencing (mNGS) analyses of nasopharyngeal aspirate and blood specimens verified an HPIV-3 infection, with negative bacterial culture results. The HPIV-3 strain detected in this patient was subtyped as HPIV C3a, and two unreported amino acid mutations were found in the HN protein region.

**Conclusion:**

The patient had a severe infection associated with HPIV-3, which was the cause of sepsis and septic shock. This study showed the diagnostic value of mNGS in etiological diagnosis, especially in severe neonatal case.

## Background

Sepsis is a systemic inflammatory syndrome triggered by a variety of pathogens (such as bacteria, viruses, and fungi), which can lead to severe sepsis, septic shock and even multiple organ dysfunctions [1]. Neonatal sepsis (NS) remains the third leading cause of mortality among neonates despite continuous improvements in neonatal medicine [2]. Although bacterial infections are the major etiological factors of sepsis, specimen detection turned out to be negative for bacteria in almost 42% sepsis patients [3]. In fact, viral sepsis was previously under-recognized due to limited diagnostic techniques for the detection and identification. Recently, a wide variety of viruses have been confirmed to cause sepsis with the application of different technology, including PCR and metagenomics next-generation sequencing (mNGS). Sepsis has been reported in children and adults caused by dengue fever virus, enterovirus, influenza virus, and herpes simplex virus [4]. A few studies have proposed that critically ill patients with severe pneumonia, acute respiratory distress syndrome (ARDS), sepsis, or myocarditis should be tested for influenza virus regardless of respiratory symptoms and epidemiology [5].

HPIVs are divided into types 1 to 4, which belong to two genera of HPIV, *Respirovirus* (HPIV-1 and HPIV-3) and *Rubulavirus* (HPIV-2 and HPIV-4) of the *Paramyxoviridae* family. Hospitalized children with acute respiratory tract infections had a high detection rate of HPIVs, ranging from 9 to 30%, only below the rate of respiratory syncytial virus (RSV) [6, 7]. Infection with HPIVs can present as asymptomatic, fever, cough, runny nose, wheezing, or apnea, with an incubation period ranging from 1 to 7 days [8, 9]. HPIVs can cause outbreaks of nosocomial infections in neonates and immunocompromised children, mainly characterized by respiratory symptoms and even respiratory failure in severe cases, and mechanical ventilation (MV) is an irreplaceable therapeutic measure for some critically ill children [10]. However, there is little evidence of HPIVs associated with neonatal sepsis.

In this paper, we report a neonatal case that showed a weak response to external stimuli, poor appetite and hematuria at onset, then rapidly progressed to septic shock and death. The etiological agent in this case was identified as HPIV-3 by mNGS method. The study suggested a potential link of neonatal sepsis with HPIV-3 and should be considered by clinicians. Meanwhile, mNGS has shown the value of clinical applications for infants with neonatal sepsis with unknown etiology.

## Case presentation

This was a G2P2 (mother: gravida 2, para 2) baby boy with 8 days of age who was born by caesarean section at full term with birth weight 3660 g. His perinatal history was un-eventful. His mother had herpes zoster at 4–5 gestational weeks and common cold at 32 gestational weeks. No family history of genetic diseases was found in his families. Neither the mother nor the infant was found any lab evidence of vertically transmitted diseases, including HIV and CMV. The boy had a healthy elder brother (3 years old).

**Fig. 1:**
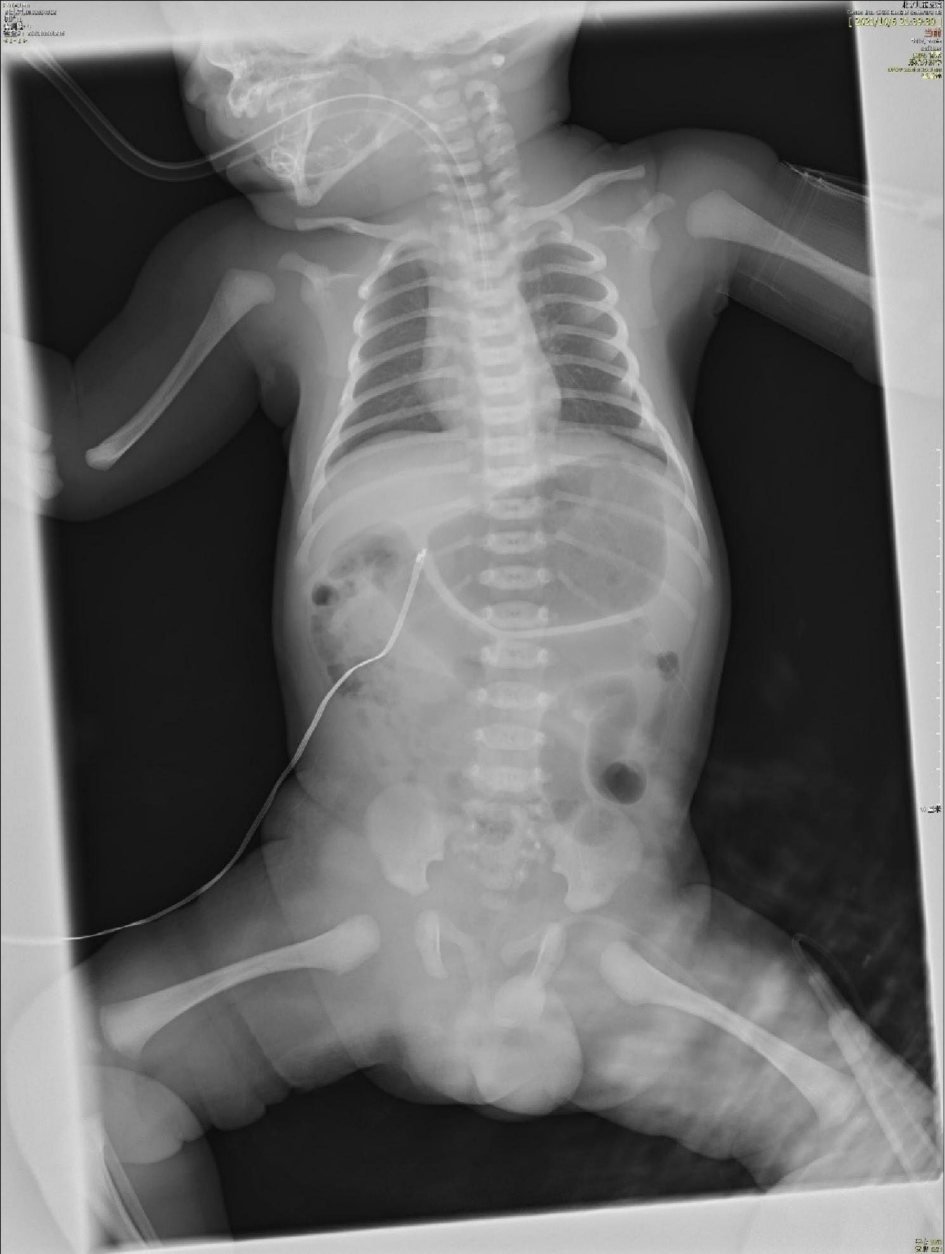
The anterior-posterior abdominal and chest X-ray in NICU. The first anterior-posterior abdominal and chest X-ray after the patient was admitted to the NICU when he was intubated with low settings of mechanical ventilation. Lungs were normal. Gastric dilatation and slight sausage-like colon were observed. No chest fluid nor abdominal fluid sign was observed. Abbreviation: NICU, neonatal intensive care unit

He started to have jaundice on his day of life 4 (DOL 04). The jaundice was slightly progressed but his condition was generally well, and no medical intervention was required. He suddenly presented gross hematuria on DOL 08 with unknown reason. Then he progressively presented gloomy spirit, inactivity and loss of appetite. There was no fever, hematochezia, ecchymosis, or petechiae. After 6 h (17:50, October 06), his parents took him to the Accident & Emergency (A&E) Department of Beijing Children’s Hospital, Capital Medical University, Beijing. He had septic shock when he arrived the A&E Department requiring immediate intubation and mechanical ventilation. Fluid resuscitation and intravenous antibiotics were then started. He was emergently transferred to the neonatal intensive care unit (NICU). His first hemoglobin (Hb) was 122 g/L when he arrived the A&E Department (18:00, October 06), but dropped to 45 g/L in 2 h (Ret 2.57%), requiring repeated packed red blood cell (pRBC) transfusion. His abdominal and chest X-ray found no specific abnormalities (shown in Fig. 1). During his stay in the NICU, he was given 2 times of pRBC (Table 1) due to refractory drop of Hb. No blood loss was found in brain, lungs, abdominal organs and intestine by bed-side ultrasound examination. A urethral catheter was inserted in the A&E Department, but no further urine was collected except for 3ml blood-look urine. His blood gas was severe refractory mixed acidosis. The coagulation test was prothrombin time (PT) 38.4s, fibrinogen (FIB) 0.5 g/L, activated partial prothrombin time (APTT) > 180s(normal range: 11–14 s, 2–4 g/L and 25–37 s, respectively), blood ammonia was 352 µmol/L (normal range: 18–72 µmol/L). He had completely negative response to all anti-shock treatments including fluid resuscitation and vasopressor supports, and died 14 h later.

**Table 1:** Blood gas, general lab tests, blood transfusion and vasopressor applications

Date	Time	Whole Blood Count			Gas			Serum K^+^ (mmol/L)	Vasopressor		Blood transfusion and others
		Hb (g/L)	WBC (×10^9^/L)	PLT (×10^12^/L)	pH	BE (mmol/L)	cLac (mmol/L)		Dopamine (g/kg.min)	Adrenaline (ug/kg.min)	
Oct 06	17:50	122*	14.1	188	-	-	-	-	-	-	Intubation**
	18:00	85	16.2	159	7.264	-9.1	6.8	-	5	-	Fluid resuscitation
	20.00	100	17.6	122	7.163	-21.3	14.7	7.5	6	0.1	FFP
	22:00	45	12.5	97	7.311	-16.6	27	-	11	0.2	
	23:00	-	-	-	-	-	-	8.6	11	0.2	washed pRBC
Oct 07	02:00	98	9.6	64	7.289	-16.6	26	7.5	15	0.2	Arginine
	03:00	-	-	-	-	-	-	-	15	0.2	FFP, calcium gluconate
	04:00	-	-	-	-	-	-	-	20	0.3	
	06:00	38	10.3	68	7.220	-13.1	HH	-	20	0.6	washed pRBC
	07:00	-	-	-	-	-	-	8.9	20	0.6	peritoneal dialysis
	09:00	39	7.4	71	-	-	-	7.3	20	0.6	
	09:37	Passed away									

*Capillary blood (the other blood tests were from arterial blood)

**Patient arrived the Accident & Emergency Department of Beijing Children’s Hospital

Abbreviations: BE, base excess; FFP, fast frozen plasma; Hb, hemoglobin; PLT, platelet; pRBC, packed red blood cell; WBC, white blood cell;

Five days after death, his blood cultures were reported negative for bacteria, but pharyngeal swab and blood were positive for HPIV-3 virus nucleic acid. Whole exome sequencing did not discover any potential single gene disease or chromosome disorders, such as coagulation abnormalities, congenital metabolic diseases and immunodeficiency diseases. There was no abnormality of copy number variation sequencing (CNV-seq).

### Nucleic acid detection by PCR

Nucleic acid testing was performed on pharyngeal swabs and serum samples from the patients. The nucleic acid was extracted using a nucleic acid extraction kit (Da An Gene, Guangzhou, China, Cat. DA0630) and automatic nucleic acid extractor instrument provided by Da An Gene Co., Ltd. The multiplex real-time PCR assay (XABT, Beijing, China) was performed by LightCycler®480 PCR instrument (Roche, Basel, Switzerland). The multiplex PCR kit for 6 respiratory pathogens, including respiratory syncytial virus (RSV), adenovirus (ADV), influenza A virus (Flu A), influenza B virus (Flu B), human parainfluenza virus 1(HPIV-1) and human parainfluenza virus 3(HPIV-3). Both pharyngeal swab and serum sample of the patient showed a positive result in the fluorescent channel of HPIV-3 (Ct values were 17.31 and 35.76, respectively), with CT value less than 36.

### Metagenomics next-generation sequencing (mNGS) for pathogen detection

Pharyngeal swabs and serum samples (collected on the day of admission) from the patients were sent to Tianjin Novogene Medical Laboratory for mNGS. The nucleic acid was extracted using QIAamp Viral RNA Mini Kit (52,906, Qiagen Biotech, Germany). RNA libraires were constructed using TIANSeq Fast RNA Library Kit (NR102, Tiangen Biotech, Beijing, China). After the removal of rRNA, RNA fragments of size 150–200 bp were obtained using the enzyme. The first strand cDNA was synthesized, and then the second strand cDNA was synthesized, followed by terminal repair and A-tailing reactions. Adapters were ligated onto the A-tailed fragments. Fragments with adapters were purified and amplified using PCR. After purifying the PCR product, the libraries were pooled and sequenced for 50 bp single ends on an Illumina NovaSeq 6000 machine. The data output of 40 M reads was obtained. Reads were then mapped against the human reference genome using Bowtie2 v2.3.5.1 [11]. After alignment, we removed human reads, the remaining reads were aligned with PD-seq^™^ database v1.0, which is a self-built database of 9,295 bacteria, 7,210 viruses, 412 fungi and 104 parasites. Kraken2 v2.1.2 [12] and Bracken software v2.6 [13] were used to further analyzed and annotated.

Due to the short time between onset and death, we did not have enough blood samples to detect mNGS, but HIPV-3 was the causative agent of this patient’ death according to the positive result by mNGS in pharyngeal swab and PCR in blood. Additionally, *Acinetobacter baumannii* (295 reads) and *Trichosporon asahii* (15 reads) were detected in pharyngeal swab, however bacterial culture of blood specimens was negative, so they are not considered to be the causal pathogens.

### Viral molecular analysis

**Fig. 2:**
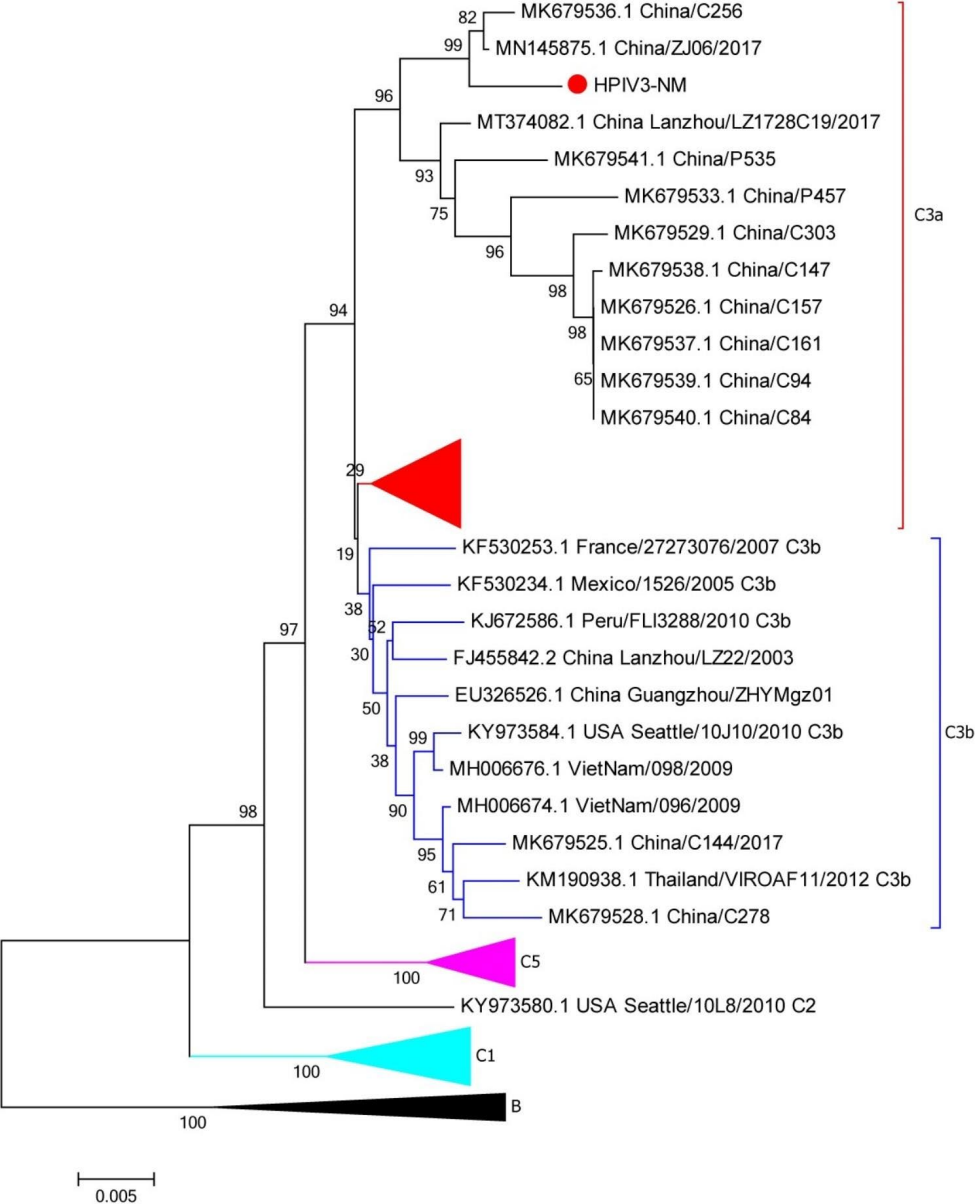
Phylogenetic analysis of HPIV-3 based on the complete genomes

The resultant strain belongs to HPIV-3 and was named HPIV3/BCH/NM (shown in Fig. 2). The whole genome sequence is retrieved by mNGS method and has been deposited in GenBank (accession number: OQ785280). Clean Data were assembled with SOAP denovo software [14, 15]. Phylogenetic analysis of nucleotide sequence based on partial polyprotein gene (HN) or complete genomes of HPIV3/BCH/NM used Neighbor-Joining method with 1000 bootstrap replicates in MEGA 7.0 program. It showed that the HPIV3/BCH/NM strain belongs to Cluster C3a, which is the most common subtype in Beijing. Nucleotide homologies analysis showed that the HPIV3/BCH/NM strain was closely related to the Human respirovirus 3 isolate C256 and HPIV3/06/ZJ/CHN/2017 from China. Comparing with the Wash/47,885/57 prototype strain of HPIV-3, Fourteen amino acid changes were identified in the HN protein region for the strain found in this study. Two unreported amino acid mutations (N86S and S512A) were found in the HN protein region. It is worth noting that S512A lie within a beta − 5 sheet of a key region for the HN protein and cell receptor binding [16].

## Discussion

Infection with HPIV-3 is initiated by viral glycoprotein-mediated fusion between viral and host cell membranes. The envelope of HPIV-3 contains two viral glycoproteins: the hemagglutinin-neuraminidase (HN) and the fusion protein (F) [17]. In addition to its receptor-attaching and fusion-promoting functions, the HN protein is also a neuraminidase that promotes the release of virions from cells and prevent them from self-agglutinating. These functions have made it a key molecule when HPIV-3 infects a host cell. Further analysis of the amino acid substitutions in the HN protein region is crucial for virus infectivity and host cell tropism.

HN is a type II transmembrane protein, which perform a number of functions by coordination with its cytoplasmic domain, membrane-spanning region, stalk region, and a globular head. The receptor binding site is located at the center of the beta-propeller in a globular head for HPIV-3. The seven highly conserved amino acid residues (R192, D216, E409, R424, R502, Y530 and E549) is crucial for combination with N -acetylneuraminic acid (Neu5Ac) of sialic acid [18]. The HPIV-3 found in this study was sequenced and found no mutations at any of these sites. N86S and S512A, previously unreported amino acid mutations, were discovered in this study. The clinical impact of this mutation in HPIV-3 needs to be confirmed by long-term follow-up outcome studies.

The yearly Infection rates of HPIV ranged from 1.9 to 12 per 1000 children younger than 1 year [19], in which most of the illnesses were upper respiratory tract infections (40-60%) and some were lower respiratory tract infection (< 20%) including pneumonia and acute bronchitis [20, 21], Human parainfluenza virus 3 (HPIV-3) is the common pathogen causing respiratory disorders in infants and young children compared to other parainfluenza viruses [22]. Newborns and young infants are particularly susceptible to HPIV infection, numerous outbreaks of HPIV have been reported in the NICU [23, 24]. After the procedure of respiratory treatment, the majority of children with HPIV-3 have a favorable prognosis, with rare fatalities [23, 25].

HPIV infection has caused other systemic diseases in some cases. HPIV-1 and HPIV-3 can cause mumps-like disease reported by Bloom et al. [25]. It was previously reported that HPIV-3 and HPIV-4 were responsible for 62% and 10% of febrile seizures, respectively, and HPIV infections were associated with meningitis, encephalitis and ventriculitis [26–28]. MacDonald et al. have reported that HPIV infection can aggravate primary nephrotic syndrome [29]. The nucleocapsid-like structures of HPIV were detected in adults and children with hepatitis [30]. Meanwhile, HPIV infections have been reported to associate with myocarditis and pericarditis [31]. Kazuhiro et al. reported a fatal case of rhabdomyolysis in a child infected with HPIV-3 [32], and James et al. reported that HIPV-2 causes myoglobinuria in an adult [33]. However, there is no report of neonatal sepsis caused by HPIV-3.

Currently, there is no effective antiviral drug available for this pathogen. Symptomatic support is the main treatment for patients with HPIV-3 infection. Some studies suggest that patients can be treated with intravenous immunoglobulin (IVIG) and glucocorticoids [34]. Notably, DAS181 is originally used as a drug to treat seasonal influenza virus infections, which can block viral binding to receptors on host cells and prevent entry [35]. In immunodeficient patients with HPIV-3 infection, it exhibits good curative effects [36, 37]. Other antiviral drugs under development include HN inhibitors, bcx2798 and bcx2855. They act by competing for the binding site of virus invasion into host cells, and were shown to be highly effective in a humanized mouse model of infection [38, 39].

Most people with HPIV infection have a good prognosis, but in immunocompromised patients, such as those with blood system diseases or those who received hematopoietic stem cell transplantation, can lead to viremia, disseminated infection and even death. Hematopoietic stem cell transplants have been reported to have a mortality rate of 10 to 33% infected with HPIV [40–42].

## Conclusion

We reported a rare neonatal death case of sepsis and septic shock caused by HPIV-3. mNGS was needed to make a rapid diagnosis of critical cases, especially in neonate. Further research is needed to understand the pathophysiology and risk factors of neonatal sepsis after HPIV infection, as well as the management of HPIV-3 mutation S512A.

## Data Availability

All data that support the findings of this study are included in this article and its online supplementary material. Further inquiries can be directed to the corresponding author.

## Abbreviations

HPIV: Human parainfluenza virus
HPIV-3: Human parainfluenza virus type-3
MNGS: Metagenomic next-generation sequencing
NS: Neonatal sepsis
ARDS: Acute respiratory distress syndrome
HPIV-1: Human parainfluenza virus type-1
HPIV-2: Human parainfluenza virus type-2
HPIV-4: Human parainfluenza virus type-4
RSV: Respiratory syncytial virus
MV: Mechanical ventilation
DOL: Day of life
A&E: Accident & Emergency
NICU: Neonatal intensive care unit
Hb: Hemoglobin
pRBC: Packed red blood cell
PT: Prothrombin time
FIB: Fibrinogen
APTT: Activated partial prothrombin time
CNV-seq: Copy number variation sequencing
ADV: Adenovirus
Flu A: Influenza A virus
Flu B: Influenza B virus
HN: Hemagglutinin-neuraminidase protein
F: Fusion protein
Neu5Ac: N -acetylneuraminic acid
IVIG: Intravenous immunoglobulin
